# Hierarchical Structure and Properties of the Bone at Nano Level

**DOI:** 10.3390/bioengineering9110677

**Published:** 2022-11-10

**Authors:** Farah Hamandi, Tarun Goswami

**Affiliations:** 1 Department of Biomedical, Industrial and Human Factors Engineering, Wright State University, Dayton, OH 45435, USA; 2 Department of Orthopedic Surgery, Sports Medicine and Rehabilitation, Wright State University, Dayton, OH 45435, USA

**Keywords:** bone tissue, calcium, collagen fibril, hierarchical, hydroxide, hydroxyapatite, mineral surface

## Abstract

Bone is a highly hierarchical complex structure that consists of organic and mineral components represented by collagen molecules (CM) and hydroxyapatite crystals (HAC), respectively. The nanostructure of bone can significantly affect its mechanical properties. There is a lack of understanding how collagen fibrils (CF) in different orientations may affect the mechanical properties of the bone. The objective of this study is to investigate the effect of interaction, orientation, and hydration on atomic models of the bone composed of collagen helix (CH) and HAC, using molecular dynamics simulations and therefrom bone-related disease origins. The results demonstrate that the mechanical properties of the bone are affected significantly by the orientation of the CF attributed to contact areas at 0° and 90° models. The molecular dynamics simulation illustrated that there is significant difference (*p* < 0.005) in the ultimate tensile strength and toughness with respect to the orientation of the hydrated and un-hydrated CF. Additionally, the results indicated that having the force in a longitudinal direction (0°) provides more strength compared with the CF in the perpendicular direction (90°). Furthermore, the results show that substituting glycine (GLY) with any other amino acid affects the mechanical properties and strength of the CH, collagen–hydroxyapatite interface, and eventually affects the HAC. Generally, hydration dramatically influences bone tissue elastic properties, and any change in the orientation or any abnormality in the atomic structure of either the CM or the HAC would be the main reason of the fragility in the bone, affecting bone pathology.

## 1. Introduction

Bone tissue contains mineral phase (70%) represented by hydroxyapatite (HAC), organic phase (30%) represented by collagen (CO), non-collagenous proteins, bone cells, and water with different percentages [1,2,3]. These contents form the complex structure and provide the material properties of the bone. Investigating the bone at the nano level (<1 µm) considers the architecture of the collagen fibrils alignment. Investigating and understanding damage development in a bone at nano level is as important as the macro and micro levels.

The bone mainly is composed of type I collagen, which as a triple helix protein has the ability to be organized into fibers that provide strength and flexibility to the bone. At the molecular level, the collagen fibril is a triple helix that includes one α2-chain and two α1-chains that are composed of glycine (GLY), proline (PLY) and hydroxyproline (HYP) [4,5,6,7]. Type I collagen can be found in other tissues such as ligaments, tendons, and skin [3]. Osteocalcin, which is another non-collagenous organic protein, has a significant role in new bone mineralization. According to Burr and Allen (Eds.) (2019, p. 127), hydroxyapatite, which is the main component of the mineral phase, is composed of calcium, phosphate, and hydroxide, where the average hydroxyapatite crystal size is 50 × 25 × 2~3 nm [4]. The mineral and collagen orientation significantly affects the mechanical properties of the bone, where bone stiffness primarily depends on the mineral phase, as the mineral density is highly correlated to the strength of the bone [4].

There are different bone diseases that affect the molecular structure of the tissue. Osteogenesis imperfecta (OI) is a genetic disorder that affects bone strength as a result of mutation in the genes (COL1A1/COL1A2) responsible for collagen type I production [8,9,10,11,12,13]. It occurs as a result of the substitution of GLY with valine, arginine, aspartic acid, glutamic acid, and cystine. This substitution affects the mechanical properties and strength of the collagen helix, collagen–hydroxyapatite interface, and eventually affects the mineral part. In general, the abnormality in the atomic structure of either the collagen or the hydroxyapatite is the main reason of the fragility in OI bone tissues.

There are several techniques that are used to characterize the orientation of the collagen fibrils. Some of these techniques are polarized Raman spectroscopy [14,15], polarized Fourier transform infrared spectroscopy [16], polarized second harmonic generation [17,18], small-angle light scattering spectroscopy [19,20,21,22], elastic scattering spectroscopy [23,24], electron transmission diffraction [25,26], electron backscatter diffraction [27], magnetic resonance imaging [28], and microwave method [29]. The main limitations with these techniques are the limited resolution, they are time consuming, costly, and lack the ability to visualize the three-dimensional orientation. Additionally, none of these techniques had the ability to provide a quantitative representation of the bone. In the current study, X-ray based technique is used to observe the bone at the nano level. The orientation of the collagen can be observed by using the X-ray based technique. A molecular dynamics simulation can be used to investigate the mechanical behavior of the bone at the nano level and the collagen helix (CH) and HAC oriented in different modes depending on the orientation observed using the X-ray-based technique.

Experimental and theoretical studies have been performed to understand the atomic structure of the bone [30,31,32,33,34,35,36,37,38,39,40,41]. Experimentally, it is still a challenge to characterize bone atomic and structural integrity at the nano level. Additionally, most studies were focused on investigating CF and fibers under tension [30,31], and investigating the effect of bone structure at nano level on bone failure [32,33]. On the other hand, the theoretical work has been performed mostly to understand the effect of the CM and HAC structure and properties on biomaterials design, such as bone scaffold hierarchical design [34,35,36,37]. A molecular dynamics simulation was exploited to investigate the variation in the material properties of the bone at the molecular level assemblies [38,39,40], and how changing the residue sequence of the collagen helix affects the molecular mechanical properties of the bone [41], and how hydration significantly affects the bone at nano level [42]. To the best of our knowledge, there is a lack in understanding the mechanical properties of the bone at the nano level with respect to CO and HAC orientation. HA surface, hydration, and the chemical environment are important features that need to be investigated. The objective of this study is to elucidate the unique contributions that nano-modeling of the bone provides in the framework of collage orientation and the effect on the bone mechanical properties. The CO-HAC interface is of fundamental importance in investigating bone under healthy and pathological status. In this study, an attempt was made to create a new model for the collagen fibrils of the bone from Swiss Light Source (SLS) data, provided by the Paul Scherrer Institute (PSI) [6], that maps the collagen order and to understand the change in the architecture of the CF and HAC configuration with damage propagation. The ADF 2019.3 modeling suite was used to perform the molecular dynamics simulation.

## 2. Materials and Method

SLS data was used to map the collagen order; then a computational three-dimensional modeling was performed using MIMICS 22.0 (Materialise—Software Mimics Innovation Suite, Leuven, Belgium) program. A molecular dynamics 3D simulation was performed to investigate the effect of the interaction, orientation, and hydration of the atomic models of the bone composed of CH and HAC. Finally, numerical analysis was performed to investigate the impact of various factors on the mechanical properties of the atomic models of the bone composed of collagen and hydroxyapatite. The statistical analysis was performed using SAS software (SAS Institute Inc. 2015. SAS^®^ 12 JSL Syntax. SAS Institute Inc., Cary, NC, USA) and JMP (SAS Institute Inc., Cary, NC, USA). One-way ANOVA test was applied in the analysis. The three factors that were considered in the analysis were the collagen fibril orientation, mineral surface, and hydration. Modulus of elasticity (*E*) was calculated using MATLAB R2021b (The MathWorks Inc., Natick, MA, USA). 

### 2.1. Computational 3D Modeling at Nano Level

**Figure 1:**
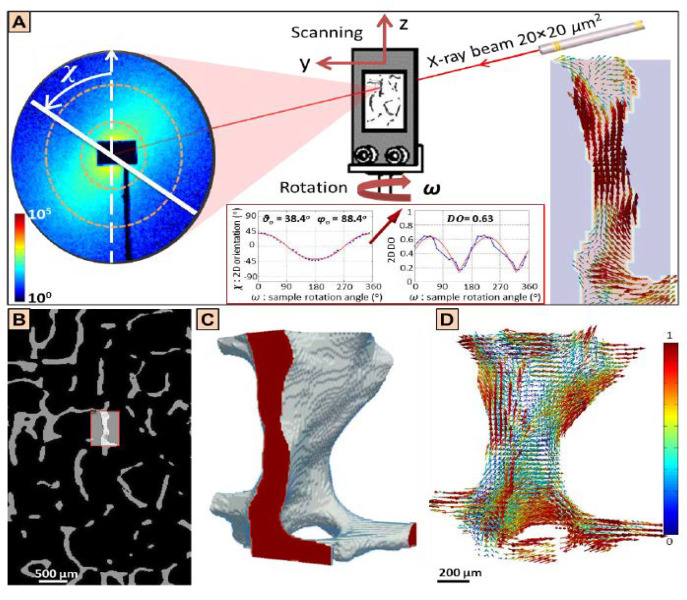
Nano-modeling for the collagen fibrils of the bone using 3D scanning small-angle X-ray scattering. (**A**) The two-dimensional mapping of the 3D organization of the collagen fibrils, (**B**) The region of interest of the trabecular bone, (**C**) The three-dimensional structure of that bone, (**D**) The three dimensional scanning small-angle X-ray scattering information [45].

**Figure 2:**
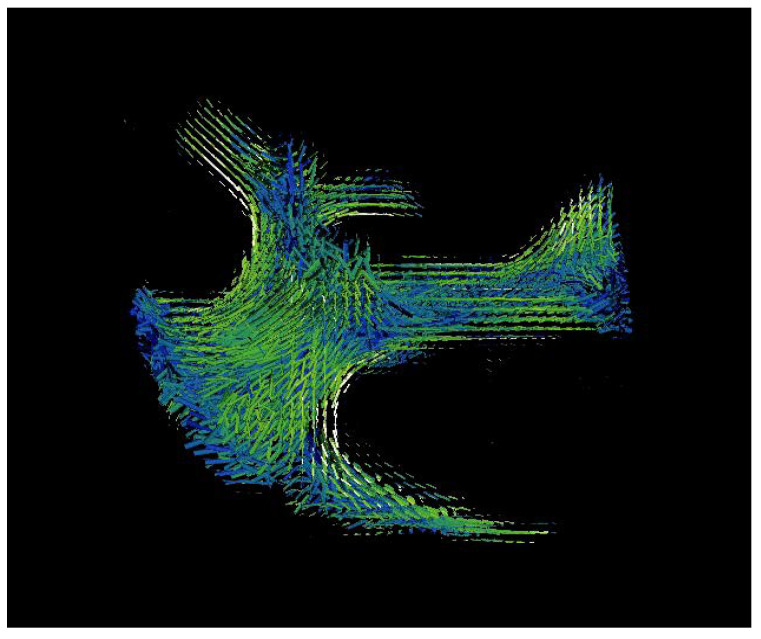
Mapping the order and alignment of the CF for a small bone sample (1 × 1 × 2.5 mm^3^) from human vertebra using Mimics.

**Figure 3:**
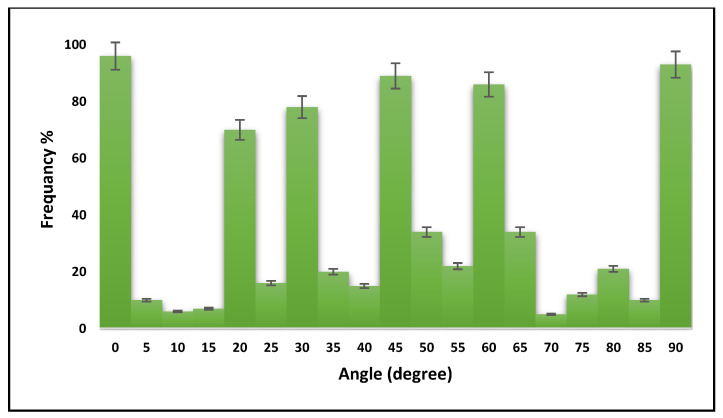
The frequency of collagen fibrils orientation angles.

SLS technology uses three-dimensional scanning small angle X-ray scattering, as shown in Figure 1. The images that were used were for a small bone sample (1 × 1 × 2.5 mm^3^) extracted from a T12 human vertebra of a 73 year old man, shown in Figure 2. The ImageJ program was used to calculate the frequency of different orientation angles, and those were (0°, 20°, 30°, 45°, 60° and 90°). Figure 3 illustrates the defining of the material properties of the bone at nano level depending on the orientation of CF. Highly-oriented vs. weakly-oriented fibrils provide anisotropic or isotropic material properties, respectively. On the other hand, previous studies proposed isotropic material properties for the bone at the micro and nano levels [43,44]. The atomic models of CH, HAC, and bone comprised of CH and HAC were modeled.

### 2.2. Molecular Dynamics Simulation (MDS)

**Figure 4:**
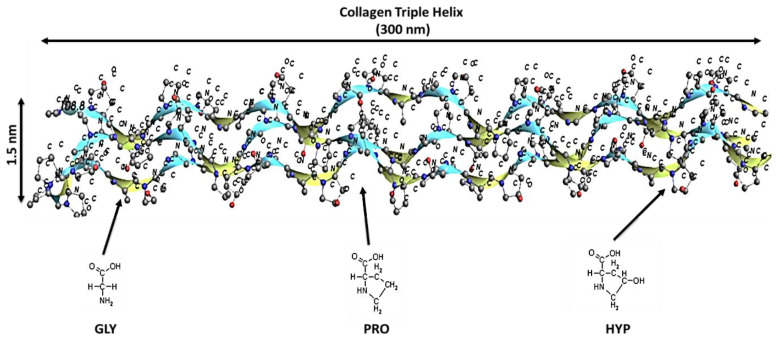
Bonds and valence angles of amino acids of collagen helix, where GLY, PRO, and HYP are glycine, proline, and hydroxyproline, respectively.

**Figure 5:**
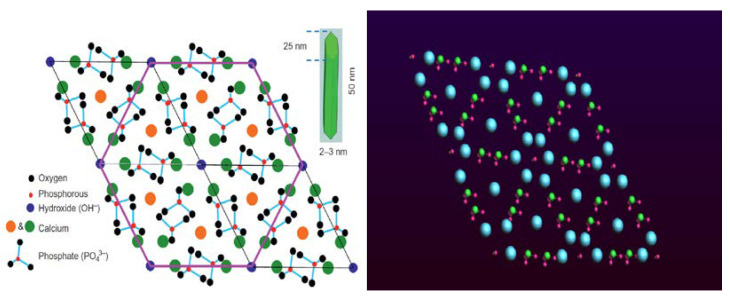
Structure of HAC [Reprinted with permission from Ref. [4]. 2019, Elsevier Science] (**left**). HA including calcium, phosphate and hydroxide modeled in ADF program (**right**).

In this study, a molecule of CO-HA composite simulation is presented, and a series of simulations were carried out to investigate the mechanical properties of the bone at nano level. Our attempt was to design a realistic representation of bone and expose it to uniaxial tensile loading. We investigated the properties of CO-HA interface and analyzed the deformation associated with both hydrated and un-hydrated conditions. Our model aimed to be as accurate as possible a representation of bone tissue at the atomic level, taking into consideration the influence of the nano level on upper micro and macro level mechanical behavior, in order to understand the complex structure of the bone. Figure 4 and Figure 5 show the structure of collagen triple helix and hydroxyapatite, respectively.

### 2.3. Forcefield

In order to perform the simulation, we developed a forcefield file that includes a previously used HA forcefield [46,47] combined with a CHARMM22 forcefield [48]. The CHARMM forcefield was used previously and has shown it is an adequate representation for the behavior of the bone at the atomic level [49,50,51,52,53]. All the molecular dynamics simulations were performed in an ADF package that has the capabilities to simulate enormous biomolecules. Van der Waals parameters were used to describe the CO-HA interaction. Equation (1) [47] was used to define the potential energy:

*E* = *E*_INTRA_ + *E*_INTER_ (1)

where

(2) $$E_{\mathrm{INTRA}}=\sum kb{\left(ri\;j-ro\right)}^{2}\;+\sum k\theta \;{\left(\theta i\;jk-\theta o\right)}^{2}+\sum k\mathrm{UB}{\left(rik-ro\right)}^{2}+\sum \left|k\varphi \right|-k\varphi \;\cos{\left(n\varphi \right)}^{2}\;+\sum k\omega {\left(\omega -\omega o\right)}^{2}$$

and

(3) $$E_{\mathrm{INTER}}=\sum \frac{1}{4\pi \varepsilon_{o}}\frac{q_{i}q_{j}}{r_{i,j}}+\sum \varepsilon_{ij}\left[{\left(\frac{R_{min,i,j}}{r_{i,j}}\right)}^{12}-2{\left(\frac{R_{min,i,j}}{r_{i,j}}\right)}^{6}\right]$$

The definitions of all the parameters are shown in Table 1.

Each CH is composed of three chains and 1014 residues, while each HA unit cell is composed of forty-four atoms with the lattice parameters shown in Table 2. Different orientations are proposed in the current study. The chain sequence of α_1_ and α_2_ type 1 collagen is shown in Table 3. These orientations are defined with respect to SLS results of collagen orientation. Each HA crystal has the same dimension and geometry, where its height is 1.6 nm. Additionally, each collagen helix length is 14.9 nm, and 1.5 nm in diameter.

To develop the model, each HA unit cell was duplicated 20, 2, and 4 times in the X, Y, and Z directions, respectively. The literature proposed that interaction with different planes of HA plays a significant role in the morphology of the material [54]. To further understand the CO-HA surface interaction, we investigated the effect of CO vs. OH planes on the total mechanical properties of the bone at nano level and how much difference the interaction plane can cause. The interface of the nano constituents was modeled. Python code was used to extract the stress–strain data for each simulation. Once the stress–strain curve was plotted, the mechanical properties were calculated. The collagen helix and HA were oriented in different modes and the simulation was performed to investigate the effect of orientation on the bone mechanical properties. Bone damage was investigated by introducing bone disease (OI) to the model, where the GLY amino acid in the collagen helix was substituted with valine, arginine, aspartic acid, glutamic acid, and cystine. We also introduced a crack with different geometries. In both methods, the mechanical properties were calculated.

## 3. Results and Discussion

**Figure 6:**
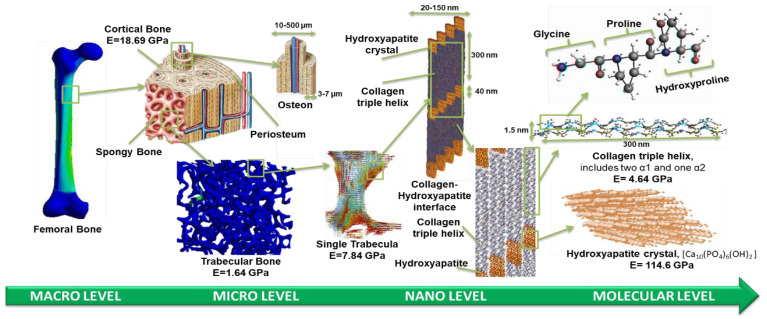
The hierarchical structure of the bone starts at the femoral bone macrolevel [55] and ends with the nanostructure and molecular level.

The current research focuses on understanding how the change in collagen fibrils orientation can improve or deteriorate the structure of the bone. The MDS revealed that having different orientations of CH and HA significantly (<0.001) affect the mechanical properties of the bone. Figure 6 presents a schematic of the bone structure at each hierarchical level, illustrating the change in the modulus of elasticity.

**Figure 7:**
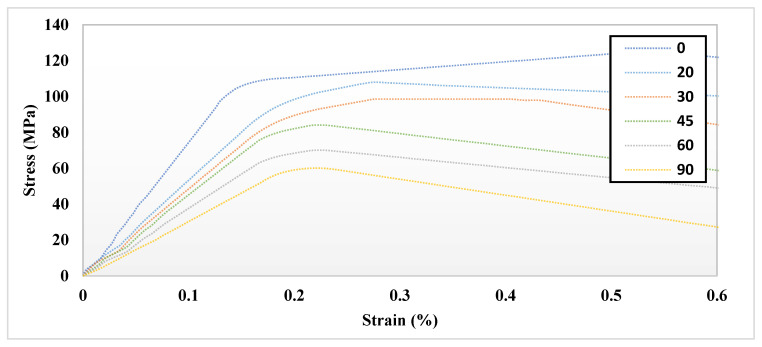
Stress–strain curve of un-hydrated collagen fibril with different orientations simulation curves (moving window average) showing the mathematical representation.

**Figure 8:**
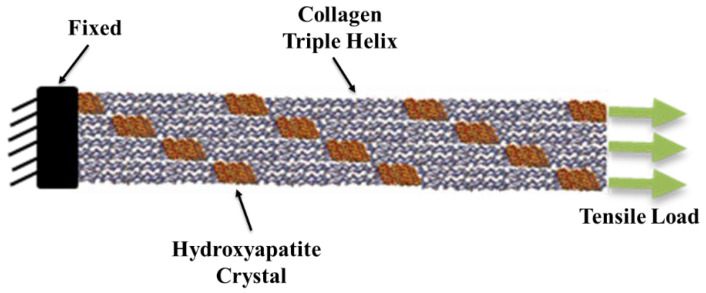

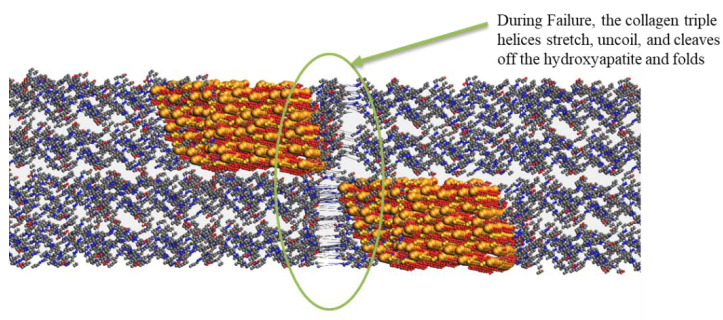
The molecular structure of the CF at the initial (**top**) and failure stages (**bottom**). Uniaxial tensile load was applied on the CF while the other end was fixed.

**Figure 9:**
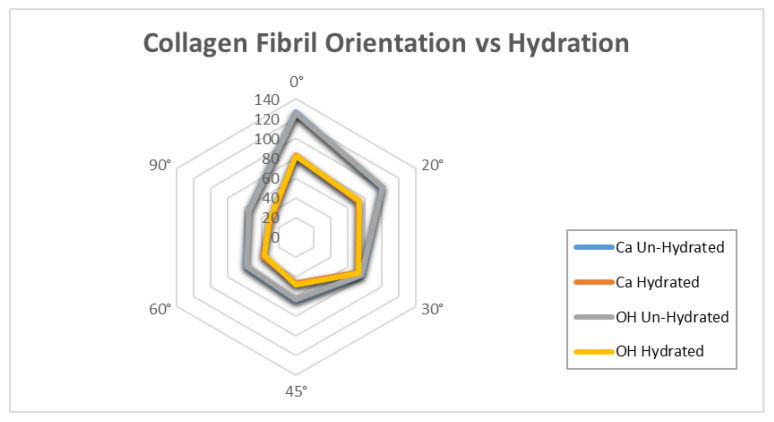
A sketch illustrates the relation between hydration, un-hydration, and collagen fibril orientations.

**Figure 10:**
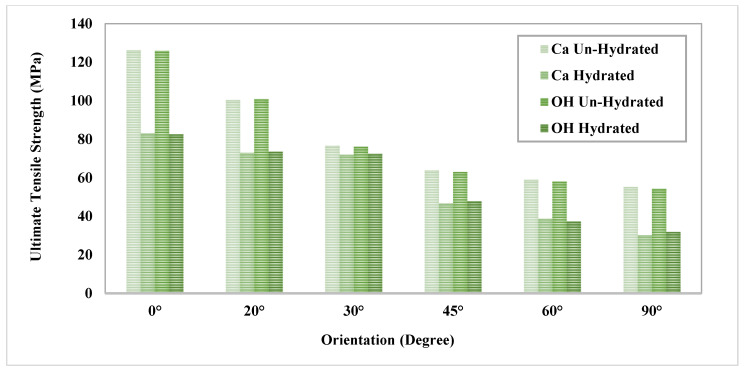
Collagen fibril ultimate tensile strength with different orientations.

**Figure 11:**
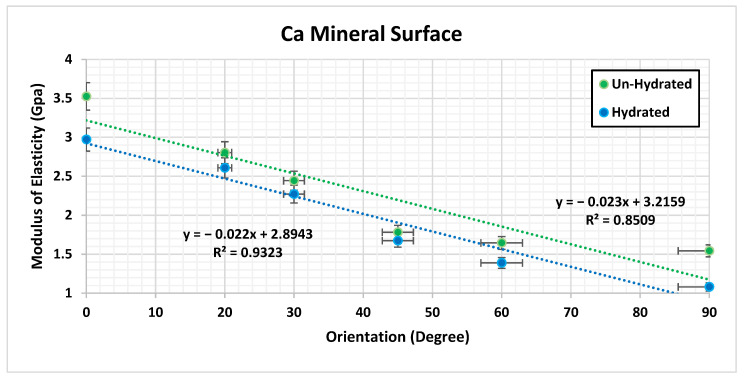
Collagen fibril modulus of elasticity with different orientations having calcium (Ca) as the mineral surface.

**Figure 12:**
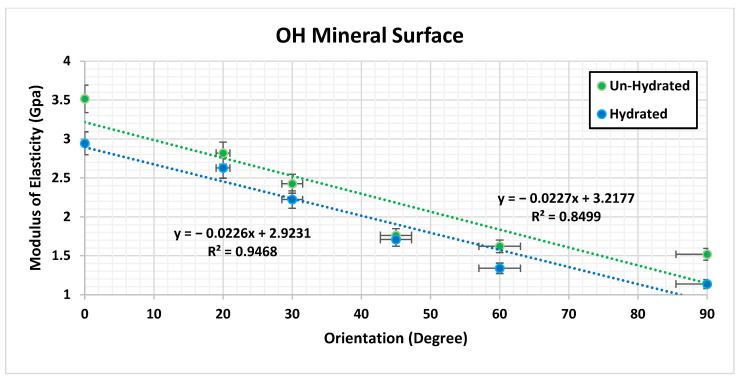
Collagen fibril modulus of elasticity with different orientations having hydroxide (OH) as the mineral surface.

The MDS illustrated that there is a significant difference (*p* < 0.005) in the ultimate tensile strength (UTS) and *E* with respect to the orientation of the hydrated and un-hydrated CF, as shown in Figure 7. The results show that having the force in a longitudinal direction (0°) provides more strength and toughness compared with the CF in the perpendicular direction (90°). The effect of hydration was observed, and the results showed that the stiffness of the CF increased in un-hydrated models. This means that the loss of water increases the rigidity of the CF and eventually makes the bone more susceptible to fracture. Figure 8 shows the molecular structure of the CF at the initial and failure stages. In general, the simulation illustrated that hydration (tightly bound water) decreases the ultimate tensile strength (UTS), toughness, and *E* of the CF. The MDS points to a loss of water in the collagen causing an increase in the UTS of bone. The simulation illustrated that there is a significant difference (*p* < 0.005) in UTS with respect to the orientation of the hydrated and un-hydrated collagen fibrils, as shown in Figure 9. However, there was no significant difference (*p* > 0.05) in the results with respect to the calcium vs. hydroxide mineral surfaces, as shown in Figure 10. The simulation demonstrated that the modulus of elasticity (*E*) calculated from the slope of a linear fit of the stress–strain curves (Table 4). The results showed that *E* was the was higher in un-hydrated simulations (2.29 ± 0.78 GPa) when compared with the hydrated simulations (1.99 ± 0.74 GPa). The results indicated that *E* was the highest in the longitudinal direction (3.53 GPa) when compared with the CF in the perpendicular direction (1.54 GPa). However, having different mineral surfaces showed no significant difference in *E* (*p* > 0.05), as shown in Figure 11 and Figure 12. The constitutive equations of modulus of elasticity with respect to collagen fibril orientation is as follows:

with OH mineral surface:

E=−0.0227×θ+3.2177            For Un-hydrated CF

E=−0.0226×θ+2.9231            For Hydrated CF

with Ca mineral surface:

E=−0.023×θ+3.2159            For Un-hydrated CF

E=−0.022×θ+2.8943            For Hydrated CF

**Figure 13:**
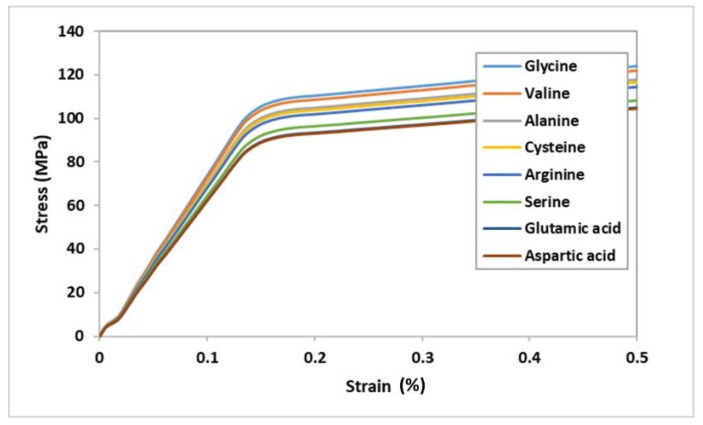
Stress–strain curve of collagen fibril substituting GLY with other amino acids.

**Figure 14:**
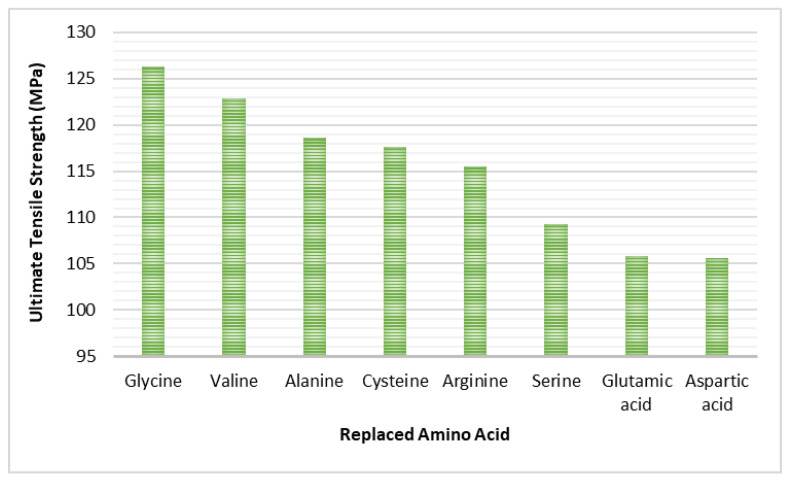
Collagen fibril ultimate tensile strength substituting GLY with other amino acids.

The MDS with proposed OI bone disease revealed that having different amino acids in the collagen helix significantly (<0.001) affects the mechanical properties of the bone. The MDS illustrated that there is a significant difference (*p* < 0.005) in the ultimate tensile strength and toughness with respect to different amino acids. The results show that substituting GLY with any other amino acid affects the mechanical properties and strength of the CH, collagen–hydroxyapatite interface, and eventually affects the HAC, as shown in Figure 13 and Figure 14. In general, the abnormality in the atomic structure of either the collagen or the hydroxyapatite is the main reason of the fragility in OI bone tissues.

### 3.1. Validation with Experimental Work

**Figure 15:**
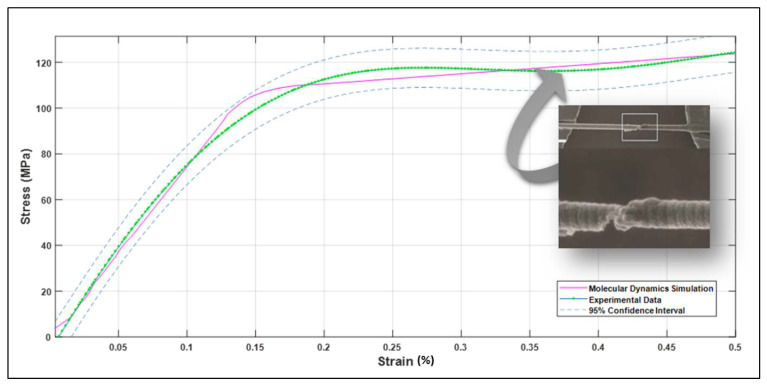
Stress–strain curve of un-hydrated collagen fibril with longitudinal orientation simulation curve (pink line). Stress–strain average curve of collagen fibril tested experimentally by Liu, Y. et al. [Reprinted with permission from Ref. [57]. 2015, Royal Society] (green line). The 95% confidence interval (dashed blue lines).

**Figure 16:**
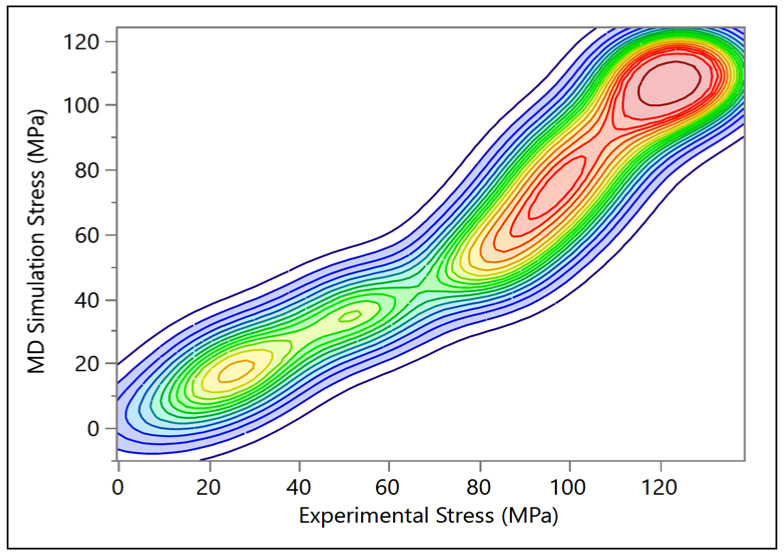
Bivariate fit of molecular dynamics (MD) simulations stress by experimental stress.

**Figure 17:**
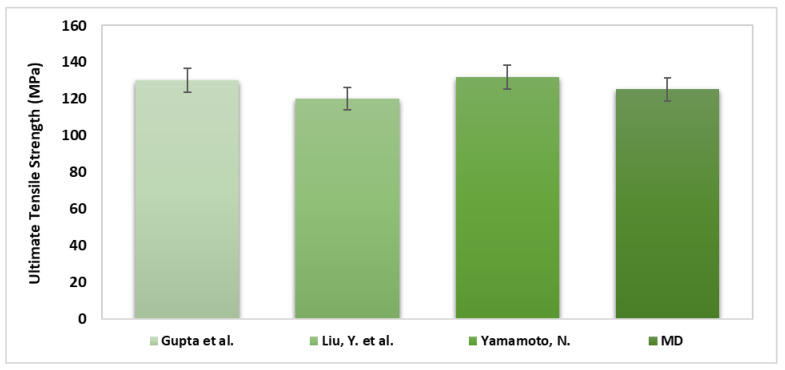
Collagen fibril ultimate tensile strength results show an agreement between the molecular dynamics (MD) simulation and previous experiment results performed by Gupta et al. [56], Liu, Y. et al. [57], and Yamamoto, N. [58].

A comparison between the MDS results and previous experimental results was evaluated. Gupta et al. [56] investigated 36 bone samples (29 wet samples and 7 dry samples). The dimension of each sample was 50 µm × 150 µm × 3 mm, and a tensile test was performed on all the models. The comparison shows agreement between the current MDS results and the experimental testing, as the dry samples showed higher modulus (13.9 ± 3.4 GPa) than the wet samples (11.5 ± 3.7 GPa). Note that the MDS show relatively good correlation with the results obtained by Liu, Y. et al. [57], where they investigated 12 samples with 305 ± 79 nm diameter and 12.7 9 ± 8.1 µm length, as shown in Figure 15 and Figure 16. The simulation was compared with Yamamoto, N. [58] experimental results. In this study, ten samples with 410 ± 60 nm were investigated. As can be seen from Figure 17, there was an agreement between the simulation and the previous experiment results in terms of ultimate tensile strength.

The numerical analysis was performed to understand the impact of various factors on the mechanical properties of the atomic models of the bone composed of collagen and hydroxyapatite. The three factors that were considered in the analysis were the collagen fibril orientation, mineral surface, and hydration. The F-test from the ANOVA indicates that the *p*-value < 0.05 and it is small and close to zero. This means that there is evidence to reject the claim that the ultimate tensile strength is similar for all the factors and at least one of them is different. The analysis showed that the collagen fibril orientations have a significant effect (*p* < 0.0001) on the ultimate tensile strength of the atomic models of the bone, and the LSMeans (least square means) differences Student’s *t*-test connecting letter report showed that the ultimate tensile strength is significantly different in terms of orientation. Hydration showed a significant effect as well (*p* < 0.0001), where un-hydrated models ultimate strength was significantly higher than hydrated models. The LSMeans differences Student’s *t*-test connecting letter report showed no significant difference between Ca and OH mineral surfaces. In general, the statistical analysis showed the collagen fibril orientation and the hydration have significant effects on the ultimate tensile strength, but the mineral surface does not have any significant effect.

### 3.2. Prediction Equation

A prediction equation was developed to predict the ultimate tensile strength with respect to different orientations of the collagen fibrils as follows:

$$\mathrm{Ultimate}\;\mathrm{Tensile}\;\mathrm{Strength}\;\;\;\;\;\;\;\;\;\;\;\;\;\;\;\;\;\;\;\;=100.12452-1.1096903\times \mathrm{Orientation}\;\;\;\;\;\;\;\;\;\;\;\;\;\;\;\;\;\;\;\;+0.0134039\times {\left(\mathrm{Orientation}-40.8333\right)}^{2}\;\;\;\;\;\;\;\;\;\;\;\;\;\;\;\;\;\;\;\;+0.0002412\times {\left(\mathrm{Orientation}-40.8333\right)}^{3}$$

**Figure 18:**
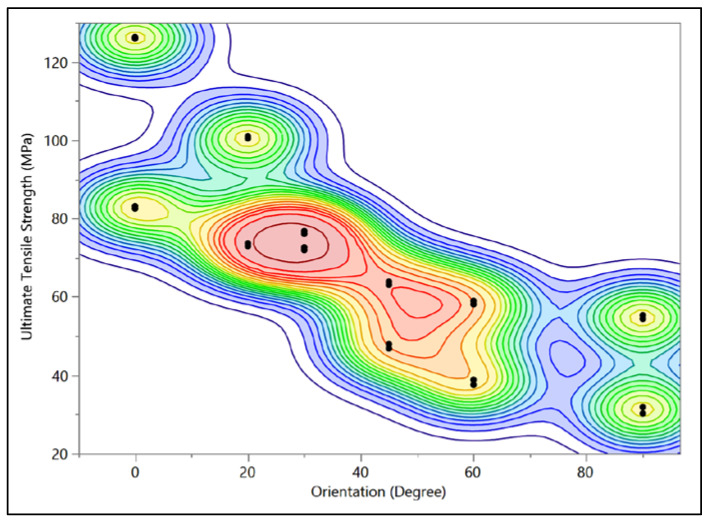
Bivariate fit of ultimate tensile strength by orientation.

Table 5 shows the prediction equations of stress vs. strain for each collagen fibril orientation. Figure 18 shows the bivariate fit of ultimate tensile strength by orientation, where the ultimate tensile strength is the highest for 0° and decreases as the orientation angle increases until reaching the minimum at 90°.

A regression model was developed for CF model ultimate tensile strength with respect to strain and the orientation angle using MATLAB.

**Figure 19:**
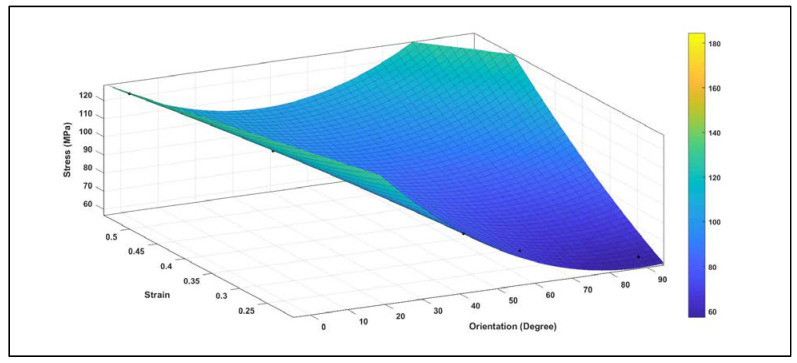
The sensitivity plot demonstrating the relationship between the maximum stress of the un-hydrated CO-HAC composite with OH mineral surface vs. the strain and the orientation of CF. The analysis illustrates that the orientation has a significant effect on stress vs. strain distribution where the stress decreases as the angle increases to reach the least at 90°.

The regression model is:

(4) $$\sigma_{U}=1.936-\left(0.4391*\theta \right)+\left(557.1*\varepsilon_{U}\right)+\left(0.03543{*\theta }^{2}\right)+\left(286.7*\theta *\varepsilon_{U}\right)-\left(8.73*\varepsilon_{U}^{2}\right)\;\;\;\;\;\;\;\;\;\;-\left(0.0005823{*\theta }^{3}\right)-\left(0.1433{*\theta }^{2}*\varepsilon_{U}\right)+\left(55.63*\theta *\varepsilon_{U}^{2}\right)-\left[\left(1.181*10^{4}\right)*\varepsilon_{U}^{3}\right]\;\;\;\;\;\;\;\;\;\;\;+\left[\left(2.72*10^{-6}\right){*\theta }^{4}\right]+\left(0.000296{*\theta }^{3}*\varepsilon_{U}\right)+\left(0.3112{*\theta }^{2}*\varepsilon_{U}^{2}\right)-\left(121.9*\theta *\varepsilon_{U}^{3}\right)\;\;\;\;\;\;\;\;\;+\left[\left(3.551*10^{4}\right)*\varepsilon_{U}^{4}\right]+\left[\left(1.954*10^{-5}\right){*\theta }^{4}*\varepsilon_{U}\right]-\left(0.008862{*\theta }^{3}*\varepsilon_{U}^{2}\right)\;\;\;+\left(1.328{*\theta }^{2}*\varepsilon_{U}^{3}\right)-\left(0.8833*\theta *\varepsilon_{U}^{4}\right)-\left(3.343e+04*\varepsilon_{U}^{5}\right)$$

where $\sigma_{U}$ is the stress in un-hydrated environment, $\varepsilon_{U}$ is the strain, and θ is the orientation angle, with SSE: $1.473\times 10^{4}\;$, R-square: 0.9324, Adjusted R-square: 0.9267, and RMSE: 8.074. This regression model can be used to predict the ultimate tensile strength of the CF with respect to different orientations. The sensitivity analysis demonstrated the relationship between the maximum stress of the un-hydrated CO-HA composite with OH mineral surface vs. the strain, and the orientation of CF obtained from the constitutive equations for each CF orientation shown in Table 5. The analysis showed the stresses were higher when CF was in the parallel direction (0°) with maximum stress (125.12 MPa). On the other hand, the stresses were the least when the CF was in the perpendicular direction (90°) with maximum stress (60.03 MPa). In general, the sensitivity analysis (Figure 19) illustrates that the orientation has a significant effect on stress vs. strain distribution where the stress decreases as the angle increases to reach the least at 90°.

## 4. Conclusions

The current investigation suggests that the mechanical properties of the bone are affected significantly by the orientation of the CF. Any change in the structure of the collagen–HAC composite would lead to variable bone diseases (OI). These results provide significant insight into the behavior of the collagen–HAC interface and represent a leap in understanding of bone material performance at the nano level. Any change in the orientation would lead to variable bone diseases. The MDS illustrated that there is a significant difference (*p* < 0.005) in the ultimate tensile strength, toughness, and modulus of elasticity with respect to the orientation of the hydrated and un-hydrated collagen fibrils. Additionally, the results show that having the force in a longitudinal direction (0°) provides more mechanical properties compared with the collagen fibril in the perpendicular direction (90°). The main reason for that difference is the fact that longitudinal orientation with 0° models had more contact areas than the perpendicular orientation with 90° models. Furthermore, validation showed agreement between the current MDS and the experimental testing. The effect of hydration was observed, and the results showed that the stiffness of the CF increased in un-hydrated models. This means that the loss of water increases the rigidity of the CF and eventually makes the bone more susceptible to fracture. Generally, the water content dramatically influences the elastic properties of the tissues. Additionally, substituting GLY with any other amino acid affects the mechanical properties and strength of the CH, collagen–HAC interface, and eventually has effects on the HAC. Consequently, it has been evidenced that the abnormality in the atomic structure of either the collagen or the hydroxyapatite is the main reason of the fragility in OI bone tissues. Further investigation is needed to understand the effect of the atomic structure and nano level mechanical properties on higher micro and macro levels of bone tissue. This study represents a step toward a deeper understanding of the structure–mechanical function relationship of the bone.

## Figures and Tables

**Table 1:** Forcefield Parameters.

Forcefield Parameters	Definition
*k*b, *kθ*, *k*UB, *kω, kφ*	Force constants
*ro*	*i* and *j* equilibrium bond length
*θ* *o*	Equilibrium bond angle
*ro*	*i* and *k* equilibrium bond length
*ω* *o*	Equilibrium improper torsion angle
*r_i_* *_,_* *_j_*	*i* and *j* distance
*r_i_* *_,_* *_k_*	*i* and *k* distance
*θ* *_i jk_*	*i, j* and *k* bond angle
*φ*	Dihedral angle
*ω*	Improper torsion angle
*q_i_, q _j_*	ith and jth particles charges
*ε* *_o_*	Vacuum dielectric constant
*ε* *_i j_*	Minimum of the van der Waals term
*R*_min_ *_,_* *_i_* *_,_* *_j_*	Zero of the van der Waals term

**Table 2:** Lattice parameters of hydroxyapatite.

Parameter	Values
a	9.4214 A°
b	2a
c	6.8814 A°
γ	120°

**Table 3:** The chain sequence of α_1_ and α_2_ type I collagen.

	The Amino Acids Sequence
**α** **_1_**	PRO HYP GLY PRO HYP GLY PRO HYP GLY PRO HYP GLY GLU LYS GLY PRO HYP GLY PRO HYP GLY PRO HYP GLY PRO HYP GLY PRO HYP GLY
**α** **_2_**	PRO HYP GLY PRO HYP GLY PRO HYP GLY PRO HYP GLY GLU LYS GLY PRO HYP GLY PRO HYP GLY PRO HYP GLY PRO HYP GLY PRO HYP

Where the amino acids: PRO, HYP, GLY, GLU, and LYS are proline, hydroxyproline, glycine, glutamic acid, and lysine, respectively.

**Table 4:** Change in the modulus of elasticity (GPa) for hydrated vs. un-hydrated collagen fibril with different orientations with respect to mineral surface.

	Ca		OH	
Orientations	Un Hydrated	Hydrated	Un Hydrated	Hydrated
0°	3.526	2.972	3.516	2.945
20°	2.804	2.608	2.819	2.628
30°	2.443	2.272	2.425	2.222
45°	1.782	1.674	1.761	1.709
60°	1.645	1.388	1.622	1.339
90°	1.543	1.080	1.519	1.137

**Table 5:** The constitutive equations for each collagen fibril orientation.

Orientation Angle (Degree)	Hydration	R-Square
	Hydrated	
0°	$\sigma_{H}$= 299029 ε^5^ − 327803 ε^4^ + 118125 ε^3^ − 17037 ε^2^ + 1333.9 ε− 8.3447	R^2^ = 0.863
20°	$\sigma_{H}$= −8E+0 ε^6^ + 9E+06 ε^5^ − 3E+06 ε^4^ + 624853 ε^3^ − 52197 ε^2^ + 1993 ε− 3.8355	R^2^ = 0.6849
30°	$\sigma_{H}$= −24472 ε^4^ + 15814 ε^3^ − 3823.3 ε^2^ + 632.3x − 4.9454	R^2^ = 0.6584
45°	$\sigma_{H}$= −30209 ε^5^ + 14289 ε^4^ − 545.52 ε^3^ − 613.53 ε^2^ + 183.59 ε− 0.4357	R^2^ = 0.6857
60°	$\sigma_{H}$= 95635 ε^5^ − 104759 ε^4^ + 37683 ε^3^ − 5406.7 ε^2^ + 421.3 ε− 2.5951	R^2^ = 0.8649
90°	$\sigma_{H}$= 49348 ε^5^ − 53425 ε^4^ + 18394 ε^3^ − 2668 ε^2^ + 330.08 ε− 1.7434	R^2^ = 0.7265
	Un-hydrated	
0°	$\sigma_{U}$= −623239 ε^6^ + 836039 ε^5^ − 418899 ε^4^ + 98072 ε^3^ − 11933 ε^2^ + 1087.6 ε− 3.508	R^2^ = 0.9771
20°	$\sigma_{U}$= −946926 ε^6^ + 1E+06 ε^5^ − 628493 ε^4^ + 143193 ε^3^ − 15405 ε^2^ + 996.93 ε− 1.2921	R^2^ = 0.8991
30°	$\sigma_{U}$= −432187 ε^6^ + 554374 ε^5^ − 264362 ε^4^ + 57925 ε^3^ − 6458.1 ε^2^ + 628.91 ε+ 0.1731	R^2^ = 0.9134
45°	$\sigma_{U}$= −431354 ε^6^ + 573149 ε^5^ − 280753 ε^4^ + 61933 ε^3^ − 5812.1 ε^2^ + 233.81 ε− 1.4616	R^2^ = 0.8757
60°	$\sigma_{U}$= −451836 ε^6^ + 619088 ε^5^ − 317077 ε^4^ + 75162 ε^3^ − 8279.7 ε^2^ + 490.36 ε− 3.6675	R^2^ = 0.9745
90°	$\sigma_{U}$= 260216 ε^6^ − 444112 ε^5^ + 270952 ε^4^ − 72211 ε^3^ + 7979.6 ε^2^ − 125.77 ε+ 2.9277	R^2^ = 0.819

## Data Availability

The data presented in this study are available upon reasonable request.
